# Beyond My Control: The Role of Sense of Control on Cognitive Functioning and Depressive Symptoms in Older Adults

**DOI:** 10.1093/geroni/igaf122.2701

**Published:** 2025-12-31

**Authors:** Sabine Lohmar, Amy Fiske

**Affiliations:** West Virginia University, Morgantown, West Virginia, United States; West Virginia University, Morgantown, West Virginia, United States

## Abstract

Depression and depressive symptoms commonly co-occur with mild cognitive impairment (MCI) and dementia in older adults (Ismail et al., 2017; Panza et al., 2010), and may occur for various reasons, including as an emotional response to cognitive decline (Bennett & Thomas, 2014; Richard et al., 2013). Emotional responses may be explained by a decline in sense of control, or the belief in one’s ability to influence their environment to achieve desired outcomes, which declines with age as cognitive and physical abilities change and limit independence (e.g., Lachman & Weaver, 1998). Using data taken from years 2006 to 2018 of the Health and Retirement Study (HRS), a mixed model regression indicated that cognitive functioning on the Modified Telephone Interview for Cognitive Status (TICS-27) was significantly associated with depressive symptoms on the Center for Epidemiologic Studies Depression (CES-D-8), β = -.02, t (45,163) = -5.65, p < .001, R2 = .49. Further, TICS-27 in 2016 predicted CES-D-8 in 2018, and this relation was mediated by sense of control in 2018, measured using the subscale of the Midlife Development Inventory, while controlling for demographic variables, β = -.02, SE = .004, 95% CI [-.03, -.01], Δ R2 = .11. The findings of this study confirm that cognitive function predicts depressive symptoms and highlight the importance of sense of control in explaining this emotional reaction. These findings have critical implications for preventing and treating depressive symptoms in older adults, particularly those with signs of cognitive dysfunction.

